# Perceptions of US Medical Students on Artificial Intelligence in Medicine: Mixed Methods Survey Study

**DOI:** 10.2196/38325

**Published:** 2022-10-21

**Authors:** David Shalom Liu, Jake Sawyer, Alexander Luna, Jihad Aoun, Janet Wang, Lord Boachie, Safwan Halabi, Bina Joe

**Affiliations:** 1 College of Medicine and Life Sciences University of Toledo Toledo, OH United States; 2 Pediatric Radiology Ann & Robert H Lurie Children's Hospital of Chicago Chicago, IL United States; 3 Department of Physiology and Pharmacology College of Medicine and Life Sciences University of Toledo Toledo, OH United States

**Keywords:** artificial intelligence, eHealth, digital health, integration, medical education, medical curriculum, education, medical student, medical school, elective course

## Abstract

**Background:**

Given the rapidity with which artificial intelligence is gaining momentum in clinical medicine, current physician leaders have called for more incorporation of artificial intelligence topics into undergraduate medical education. This is to prepare future physicians to better work together with artificial intelligence technology. However, the first step in curriculum development is to survey the needs of end users. There has not been a study to determine which media and which topics are most preferred by US medical students to learn about the topic of artificial intelligence in medicine.

**Objective:**

We aimed to survey US medical students on the need to incorporate artificial intelligence in undergraduate medical education and their preferred means to do so to assist with future education initiatives.

**Methods:**

A mixed methods survey comprising both specific questions and a write-in response section was sent through Qualtrics to US medical students in May 2021. Likert scale questions were used to first assess various perceptions of artificial intelligence in medicine. Specific questions were posed regarding learning format and topics in artificial intelligence.

**Results:**

We surveyed 390 US medical students with an average age of 26 (SD 3) years from 17 different medical programs (the estimated response rate was 3.5%). A majority (355/388, 91.5%) of respondents agreed that training in artificial intelligence concepts during medical school would be useful for their future. While 79.4% (308/388) were excited to use artificial intelligence technologies, 91.2% (353/387) either reported that their medical schools did not offer resources or were unsure if they did so. Short lectures (264/378, 69.8%), formal electives (180/378, 47.6%), and Q and A panels (167/378, 44.2%) were identified as preferred formats, while fundamental concepts of artificial intelligence (247/379, 65.2%), when to use artificial intelligence in medicine (227/379, 59.9%), and pros and cons of using artificial intelligence (224/379, 59.1%) were the most preferred topics for enhancing their training.

**Conclusions:**

The results of this study indicate that current US medical students recognize the importance of artificial intelligence in medicine and acknowledge that current formal education and resources to study artificial intelligence–related topics are limited in most US medical schools. Respondents also indicated that a hybrid formal/flexible format would be most appropriate for incorporating artificial intelligence as a topic in US medical schools. Based on these data, we conclude that there is a definitive knowledge gap in artificial intelligence education within current medical education in the US. Further, the results suggest there is a disparity in opinions on the specific format and topics to be introduced.

## Introduction

Artificial intelligence (AI) is the science of simulating human intelligence with machines for a variety of applications in all sectors, including medicine. Rapid advances in computational capabilities and cloud-based data systems, especially for the machine learning and deep learning subtypes, have led to innovative applications of AI in both clinical medicine and medical research [1-9]. For example, the CheXNeXt algorithm for chest radiograph diagnosis was found to perform at a level similar to radiologists [1]. AI algorithms can also predict future adverse medical events much better than traditional methods. One example is predicting aneurysms [4]. Even in the realm of psychiatry, AI algorithms can help detect subtle, yet key information about patients, such as speech patterns, that can predict subsequent psychosis onset [3]. For medical research applications, AI has been able to recognize complex patterns in large amounts of data (eg, gene expression and gut microbiota) to classify clinical conditions, such as cardiomyopathies [10], inflammatory bowel disease [11], and cardiovascular diseases [12]. According to Topol [6], this is beginning to have an impact at 3 levels: for clinicians, predominantly via rapid, accurate image interpretation; for health systems, by improving workflows and potentially reducing medical errors; and for patients, by enabling them to process their own data to promote health. Thus, the practice of clinical medicine is poised to drastically change with the inevitable infusion of AI.

Given the changing landscape of medical practice, a critical question is whether current medical students are being prepared during their training to effectively understand and work with AI. The intent to promote such training is evident. In the United States in 2018, the American Medical Association made it an official policy (H-480.940) to encourage medical students to understand the potential applications and limitations of AI in medicine [13]. It has been established why future doctors should study AI topics, but how and what specifically to teach has not yet been explored [13-18].

National surveys have been conducted in other countries to understand medical students’ opinions on AI in medical education; the results have outlined the potential benefits of integrating AI in medical training [19-22]. To our knowledge, 2 prior surveys have been conducted to assess perceptions by US medical students of AI and medicine. However, the first was radiology focused, and the other was based on only 1 institution [23,24]. A broad, national study has not been done. Furthermore, exploration into how US medical students want to learn about AI topics and what specific AI topics they most prefer has not been conducted [25]. Some expert commentaries have been published regarding the logistics of implementing AI topics in medicine, yet the voices of the medical students who will benefit from these implementations have not been heard [26-29]. Clearly, this is an important unmet need, especially because these students are the future physician workforce of the United States, whose work stands to be influenced by their exposure to training opportunities in AI or the lack thereof.

The current study was thus conducted to specifically examine the perceptions and interests of US medical students concerning AI and medicine. To our knowledge, this is the first nationwide survey of US medical students on this topic. Specifically, our survey was focused on (1) assessing the attitudes, knowledge, and familiarity of US medical students regarding AI in medicine, and (2) assessing the preferred media and topics of US medical students to expand their knowledge of AI as it pertains to medicine.

## Methods

### Survey Design

The survey was designed using the online app Qualtrics (Qualtrics International Inc); all the survey questions are detailed in Multimedia Appendix 1. The survey participants provided informed consent at the beginning of the survey, which had 2 main components. The first section of the survey gathered the demographics and medical education of the participants. The second section of the survey aimed to assess medical students’ perceptions and knowledge of AI and its application in medicine. The informed consent form described the survey length (5 minutes), the investigator, the purpose of the study, and the privacy policy. To limit unauthorized access, only 2 researchers could access the data. This mixed methods survey consisted of 24 multiple-choice questions on a 5-point Likert scale and a write-in section. Survey questions were generated by referring to previous, similar studies on the perceptions by medical students of AI in other countries [19-22]. Further, novel questions were added on the preferred formats for an AI curriculum in medical school. Finally, a write-in section was added that allowed for students to voice any thoughts on AI education in medicine that were not captured in the survey. There was no randomization of the order of survey questions. The premise for the length of the survey (5 minutes and 24 questions) was based on previous, similar published surveys [19-22]. Questions were presented equally on 6 webpages. Respondents could review and change their answers through a back button. Respondents were required to answer all questions, though a “not applicable” option was provided for some questions.

### Survey Distribution

Medical students across the United States were the target audience. To distribute the survey to the target audience, the deans of student affairs at all 169 US allopathic and osteopathic medical schools were contacted via email for participation. Fourteen allopathic and 3 osteopathic medical schools agreed to distribute the survey. Next, a link to the Qualtrics survey was sent to the relevant school faculty to be distributed to their students. There were no financial incentives given to the survey participants; their participation was voluntary. Overall, the survey was distributed to 11,248 students, of whom 390 responded (response rate 3.5%). A unique response ID was created on the Qualtrics survey page for each respondent to ensure that respondents did not submit the survey twice. Once completed, opening the survey link again with the same computer showed the completion page of the survey.

### Statistical Analyses

Some respondents did not answer all the questions, so we correspondingly reduced the value of N for questions that were omitted. Responses that were completed in less than 1 minute, which was determined to be the fastest time someone could realistically complete our pilot survey, were excluded. We first compared the Likert responses between respondents who had received formal AI training and those who had not. Next, we determined whether the responses on how and what the medical students wanted to learn differed between those who wanted to spend less than 3 hours per month studying AI and those who wanted to spend more than 3 hours per month. The Pearson chi-square or Fisher exact test were used depending on the context. A *P* value of <.05 was considered significant.

### Ethics Approval

This study and its anonymous online Qualtrics survey (IRB protocol 300975) was reviewed by a Social, Behavioral, and Educational Institutional Research Board Committee member at the University of Toledo. The committee member determined this study to meet criteria for exemption per 45 CFR 46.104 (d)(2)(i) or (ii).

## Results

### Demographics

Survey responses from 390 US medical students with an average age of 26 (SD 3) years were collected from 17 of the 169 US medical schools contacted (the schools are listed in Textbox 1). A total of 390 responses were received. Table 1 summarizes the demographic data of our surveyed sample. The response rate was 3.5% (390/11,248). It was not possible to calculate how many students opened the recruitment email, so we cannot report viewing or participation rates. A total of 250 of 390 students (64.1%) were from allopathic (MD) programs while 140 of 390 students (35.9%) were from osteopathic (DO) programs. The 390 participants included US medical students from all 4 years of medical education: first year (142, 36.4%), second year (94, 24.1%), third year (77, 19.7%), and fourth year (64, 16.4%). Additionally, 8 of 390 medical students (2.7%) were in the PhD component of a dual MD/PhD program. Only 34 of 390 students (8.7%) reported having received a formal education in AI topics via college courses. The median response time for the survey was 5 minutes and 18 seconds.

**Names of schools surveyed (n=17).**

- Chicago Medical School at Rosalind Franklin University of Medicine & Science
- Hofstra Northwell School of Medicine
- Medical College of Wisconsin
- Ohio State University College of Medicine
- Stony Brook University School of Medicine
- University of Central Florida College of Medicine
- University of Colorado School of Medicine
- University of Hawaii John A. Burns School of Medicine
- University of Kentucky College of Medicine
- University of Toledo College of Medicine and Life Sciences
- Virginia Commonwealth University School of Medicine
- Warren Alpert Medical School of Brown University
- Washington University in St. Louis School of Medicine
- Kansas City University College of Medicine—Joplin Campus
- Kansas City University College of Medicine—Kansas City Campus
- Ohio University Heritage College of Medicine
- West Virginia School of Medicine

**Table 1:** Demographics of survey participants (N=390).

Characteristics			Values
**Current year in medical school, n (%)**			
	First year	142 (36.4)	
	Second year	94 (24.1)	
	Third year	77 (19.7)	
	Fourth year	64 (16.4)	
	MD/PhD	8 (2.7)	
**Age, years**			
	Range	20-50	
	Mean (SD)	25.8 (3.4)	
**Prior formal AI education, n (%)**			
	No	356 (91.3)	
	Yes	34 (8.7)	

### Attitudes Toward AI in Medicine

**Figure 1:**
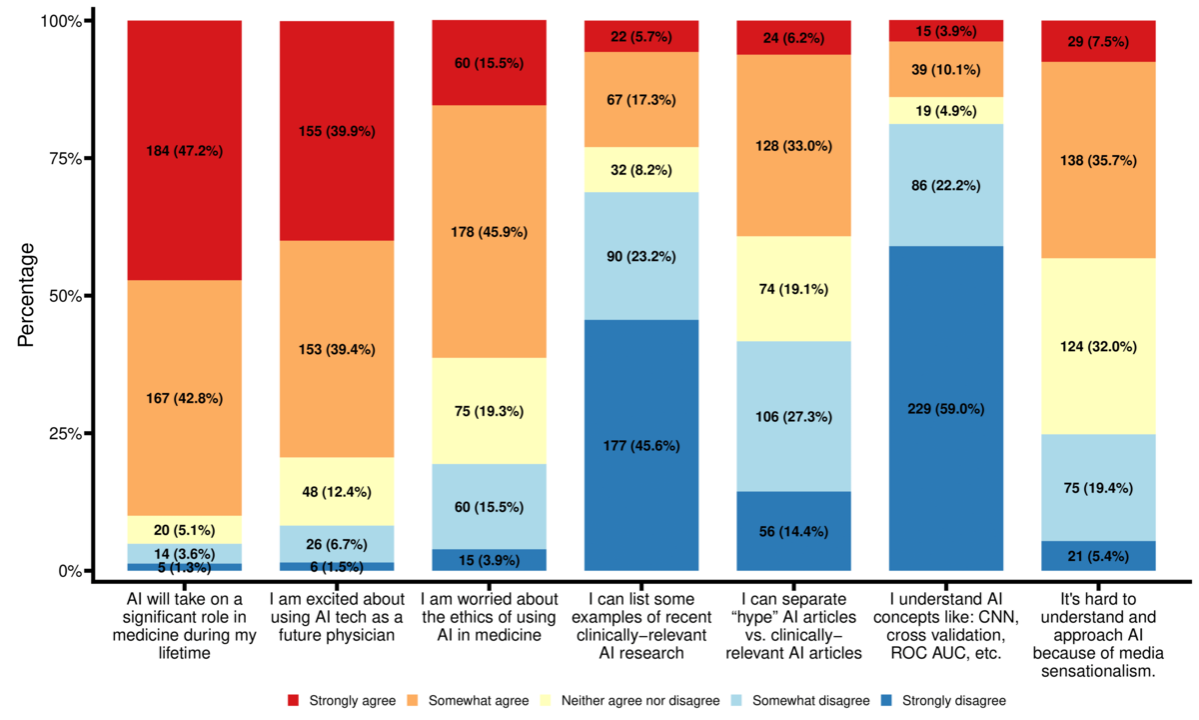
Attitudes toward and familiarity with AI in medicine of US medical students. Values indicate the number of responses (corresponding to the legend) to each statement, shown as n (%). N=388 for all statements except "AI will take on a significant role in my lifetime" (N=390) and "It's hard to understand and approach AI because of media sensationalism" (N=387). AI: artificial intelligence; AUC: area under the curve; CNN: convolutional neural network; ROC: receiver operating characteristics.

The survey assessed the general attitudes US medical students had toward AI in medicine (Figure 1). For example, 351 of 390 (90%) students agreed that AI will be a significant feature in medicine during their lifetime. Furthermore, 308 of 388 students (79.4%) were excited about using AI technology as a future physician. Despite this excitement, 238 of 388 respondents (61.3%) were broadly worried about the ethics of using AI in medicine.

The participants were prompted to select the 3 medical subspecialties they believed would be most affected by AI integration. The respondents selected diagnostic radiology (278/390, 71.3%), pathology (167/390, 42.8%), and interventional radiology (95/390, 24.6%) (Multimedia Appendix 2). Furthermore, we found that 70 of 386 (18.1%) students were less likely to enter specialties they thought would be affected by the anticipated integration of AI into that specialty (Multimedia Appendix 2).

### Knowledge of and Familiarity With AI in Medicine

Next, this survey assessed the familiarity of medical students with the application of AI in medicine (Figure 1). Only a small portion of students (54/388, 13.9%) indicated they had knowledge of core AI concepts (eg, cross validation and deep learning). Our findings further indicated that the medical students were unfamiliar with current clinical applications of AI, with only 89 of 388 (22.9%) agreeing that they could “list some examples of recent clinically-relevant AI research,” whereas 267 (68.8%) disagreed. Less than half of respondents (152/388, 39.2%) agreed that they could “separate ‘hype’ AI articles vs. clinically relevant AI articles,” whereas 162 of 388 (41.8%) disagreed. Moreover, 167 of 387 (43.2%) agreed that it was “hard to understand and approach AI because of media sensationalism,” while 124 of 387 (32%) neither agreed nor disagreed. Only 96 of 388 (24.8%) disagreed that media sensationalism made approaching and understanding AI more difficult. This survey further assessed the sources that the students had used to learn about AI in medicine; these were found to include media (263/386, 68.1%), family and friends (134/386, 34.7%), online forums (98/386, 25.4%), and professors or doctors (89/386, 23.1%) (Multimedia Appendix 2).

### Perspectives on AI in Current Medical Education Curricula

**Figure 2:**
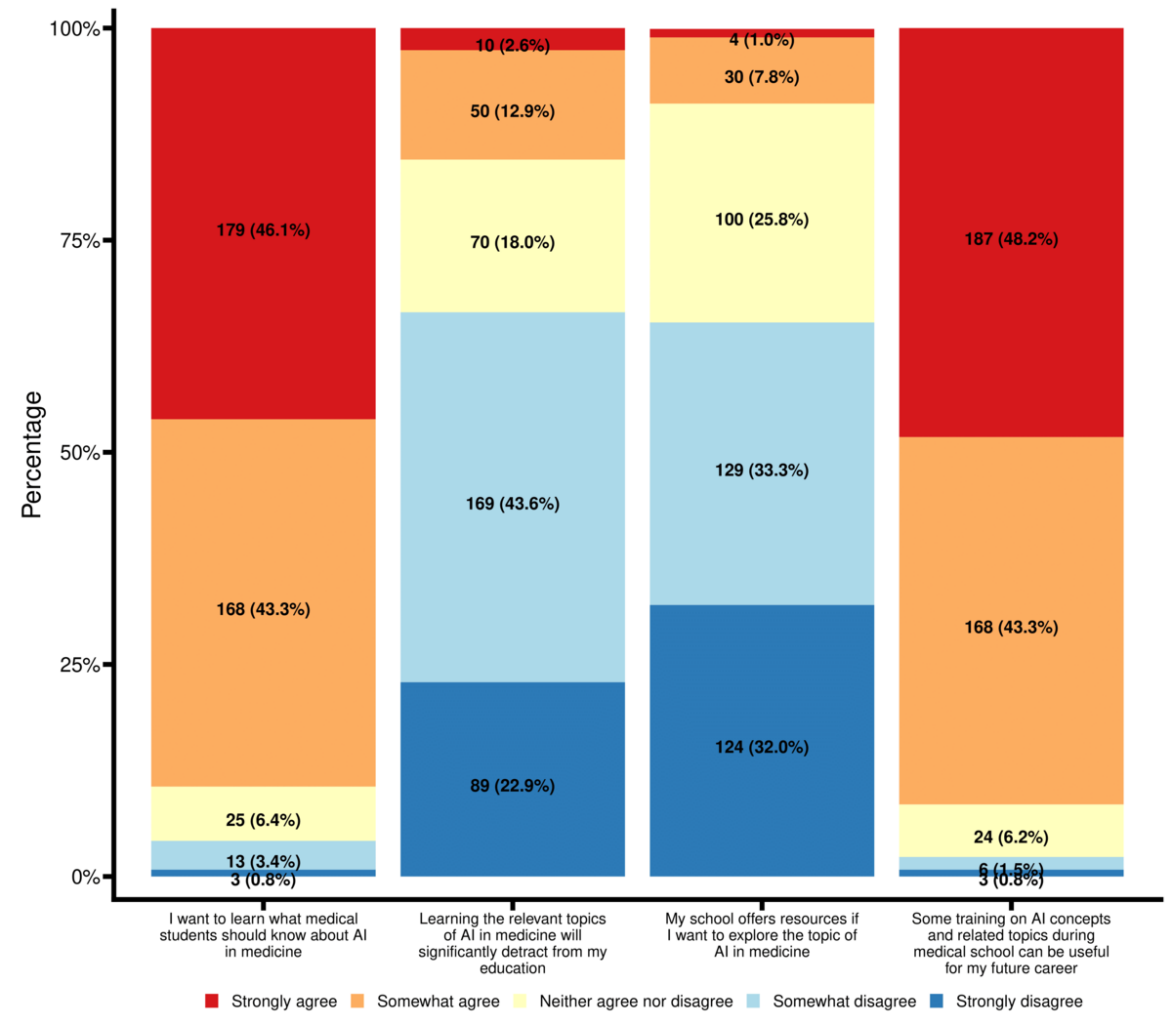
Perspectives by US medical students of AI in current medical education. Values indicate the number of responses (corresponding to the legend) to each statement, shown as n (%). N=388 for all statements except "My school offers resources if I want to explore the topic of AI in medicine" (N=387). AI: artificial intelligence.

Next, the survey assessed medical students’ perspectives on AI in current medical school education. Most students (347/388, 89.4%) agreed that they wanted to “learn what medical students should know about AI in medicine” (Figure 2). A portion of students (60/388, 15.5%) agreed that learning relevant AI topics (eg, ethics or pros and cons of AI) could significantly detract from their medical school education, while a majority of the surveyed students (258/388, 66.5%) disagreed. Despite overwhelmingly positive responses toward this topic, only 34 of 387 (8.8%) medical students agreed that their respective medical schools offered resources to explore the topic of AI in medicine. Finally, most students (355/388, 91.5%) agreed with the statement “some training in AI concepts and related topics during medical school can be useful for my future career.”

### Preferred AI Resources and Topics

**Figure 3:**
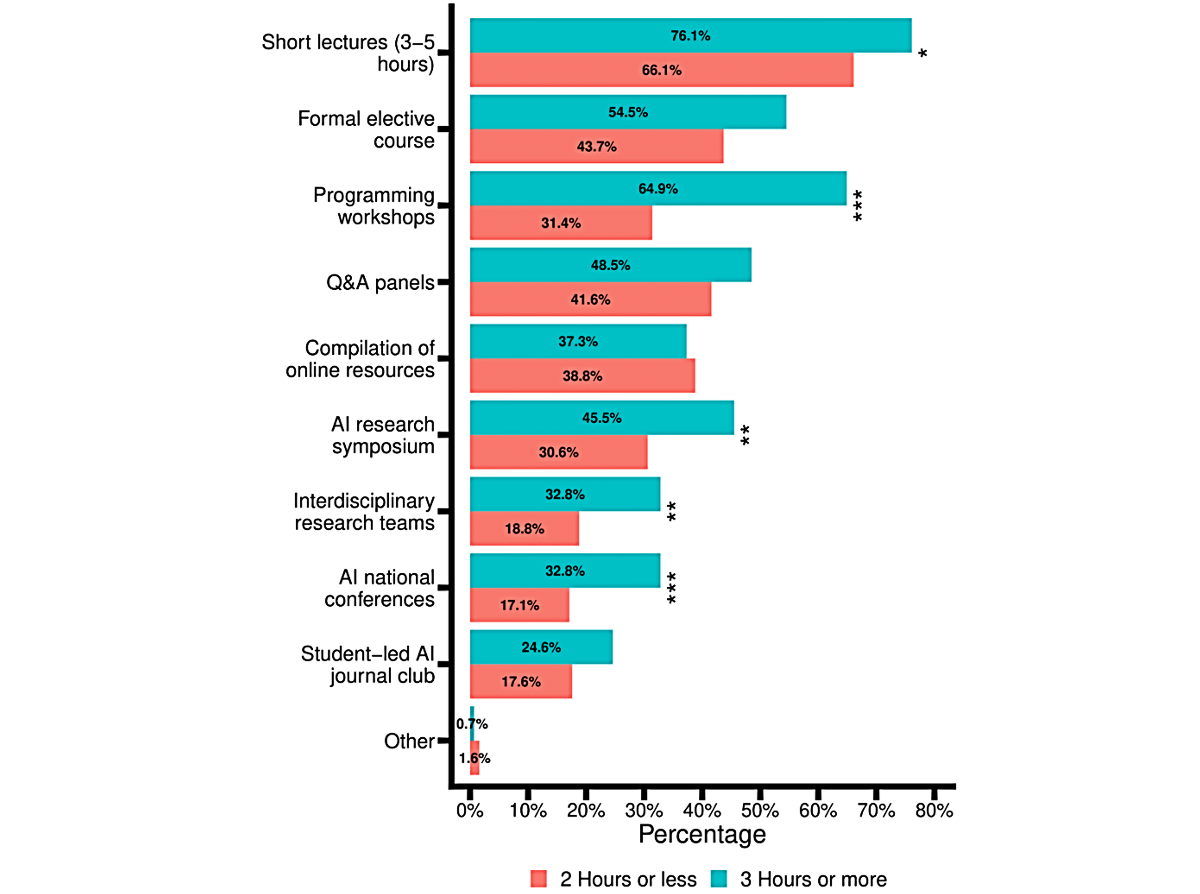
Media preferred by US medical students to explore artificial intelligence topics. Responses were classified based on how many hours a respondent had stated they would like to spend per month studying artificial intelligence in an earlier question. The red bars represent those who answered 2 hours or less and teal bars represent those who answered 3 hours or more. *P<.05, **P<.01, ***P<.001. Exact *P* values can be found in Multimedia Appendix 2. AI: artificial intelligence.

**Figure 4:**
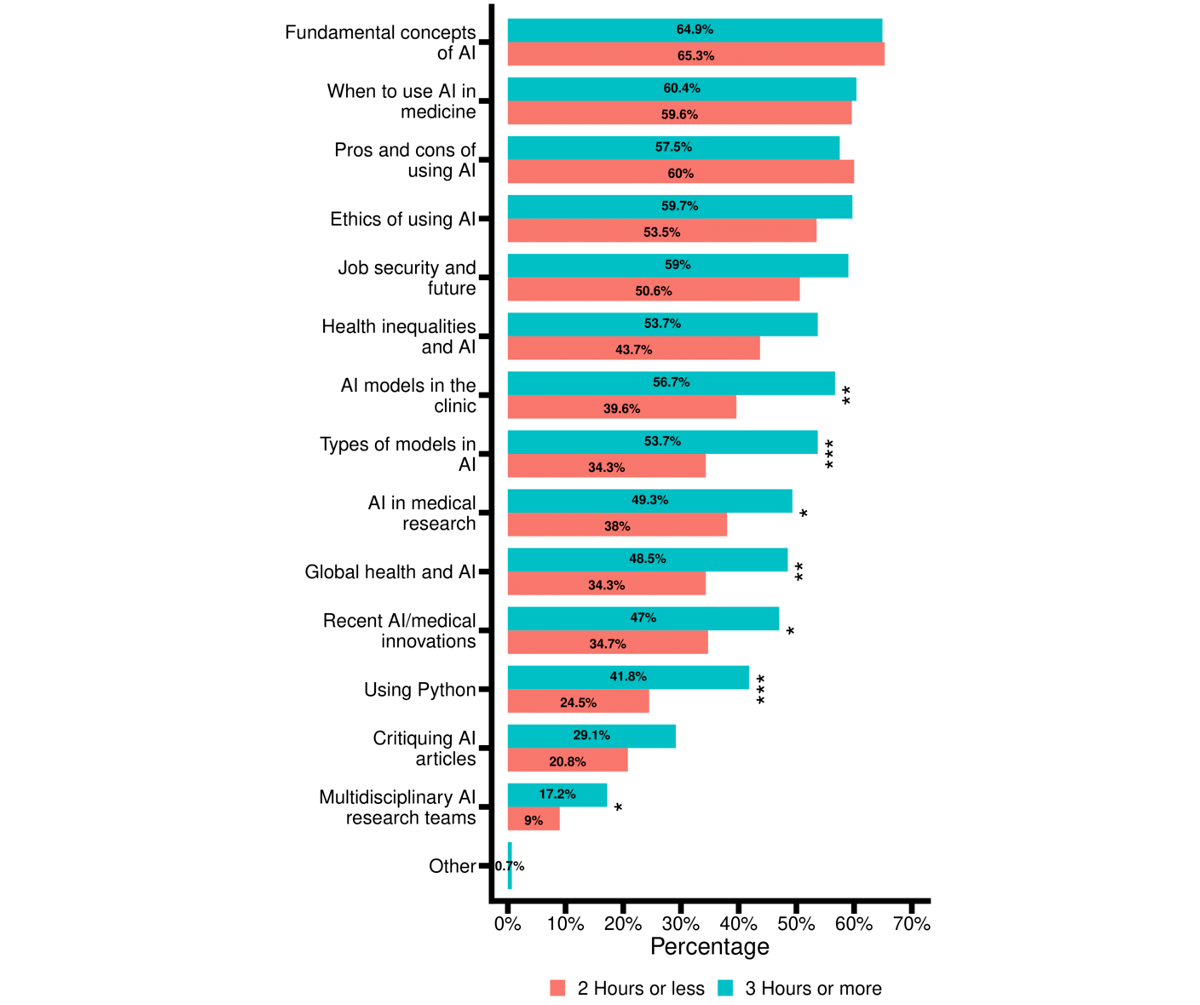
Artificial intelligence topics preferred by US medical students. Responses were classified based on how many hours a respondent had stated they would like to spend per month studying artificial intelligence in an earlier question. The red bars represent those who answered 2 hours or less and teal bars represent those who answered 3 hours or more. *P<.05, **P<.01, ***P<.001. Exact *P* values can be found in Multimedia Appendix 2. AI: artificial intelligence.

We also assessed opinions regarding AI in medical education. Over half the students (197/379, 52%) reported that 1 or 2 hours per month would be their preferred maximum amount of time spent on learning AI in medical school, whereas 123 of 379 (32.5%) students preferred that more than 3 hours per month be spent on exploring this topic and 11 of 379 (2.9%) preferred no time at all (Table 2). When the students were asked to select the resources or formats that would be most useful to learn AI in medicine, their 3 most-selected choices were short lectures (264/378, 69.8%), formal preclinical electives (180/378, 47.6%), and Q and A panels (167/378, 44.2%) (Figure 3). The medical students reported that the AI-related topics they were most interested in were “fundamental concepts of AI” (247/379, 65.2%), “when to use AI in medicine” (227/379, 59.9%), “strengths and weaknesses of using AI in medicine” (224/379, 59.1%), “ethics of AI” (211/379, 55.7%), and “what aspects of a physician’s job can be replaced with AI and which can’t” (203/379, 53.6%) (Figure 4).

**Table 2:** Time per month US medical students would prefer to study artificial intelligence topics (N=379).

Time preferred	Responses, n (%)
None	11 (2.9)
30 minutes	48 (12.7)
1 hour	100 (26.4)
2 hours	97 (25.6)
3 hours	43 (11.4)
4 hours	41 (10.8)
5 hours or more	39 (10.3)

When comparing the responses of those who preferred to spend ≤2 and ≥3 hours per month to learn AI with a chi square analysis, we found significantly different responses. Compared to the ≤2 hours per month group, the ≥3 hours per month group was more interested in short lectures (76.1% vs 66.1%, *P*=.06), programming workshops (*P*<.001), AI research symposia (*P*=.01), interdisciplinary research teams (*P*=.1), and national conferences on AI (*P*<.001). Compared to the ≤2 hours per month group, the ≥3 hours per month group was more interested in “AI models in the clinic” (*P*=.01), “types of models in AI” (*P*<.001), “AI in medical research” (*P*=.04), “global health and AI” (*P*=.01), “recent medical innovations” (*P*=.03), “using python” (*P*<.001), and “multidisciplinary AI research teams” (*P*=.03).

### Write-in Responses

A total of 23 free responses were collected (Multimedia Appendix 2, Table S4). The responses were collected and grouped into 3 categories: generally positive, academic concerns, and ethical concerns.

Several respondents emphasized the need for increasing medical students’ awareness of the role of AI in medicine. Examples include the following: “This is an extremely important topic that needs more focus,” “I honestly have heard very little about the subject,” and “I feel like I really didn't know at all about AI in medicine and hope there will be educational opportunities in the future.”

Other respondents expressed their concerns about incorporating this topic into the medical curriculum: “I am against adding more components to preclinical medical education...” and “I don't think medical students have enough computer science and engineering background to learn much about AI.” Another student noted, “It isn't terribly necessary for medical students to fully grasp all the fundamentals of AI nor for them to have programming workshops...[However,] it would be a great disservice for people walking into the field to be unfamiliar with the implications and applications of AI.”

## Discussion

### Perceptions by US Medical Students of AI in Medicine

The current study was conducted to document perceptions by current US medical students of AI and the implemention of AI knowledge into medical education. In agreement with similar survey reports from other countries [19-22], our study found that 89.4% (347/388) of surveyed US medical students wanted to learn about AI in medicine and agreed that AI would play a significant role in medicine during their future professional lives as physicians. These views did not significantly differ between those who received formal AI training and those who did not. Overall, our study supports the conclusion that current medical education in the US lags behind the enthusiasm of medical students to learn about AI with appropriate learning resources. As AI becomes continually integrated into medicine, our survey indicates that US medical education for future physicians would benefit from the addition of educational components on AI in medicine. Overall, our findings are consistent with similar survey reports from other countries. Previous survey studies showed that 70% of German, 83% of South Korean, and 78% of UK medical students agreed that AI should be part of medical training [19,21].

Although our survey suggests that 89.4% (347/388) of US medical students would like to learn about AI, only 13.9% (54/388) indicated that they understood fundamental AI terms and concepts (Figure 1). This discrepancy has also been observed in medical students in other countries [19,21]. A potential reason for this is the lack of relevant AI resources and expertise in medical education [28]. It is evident that health care in the 21st century will continue to evolve into an interdisciplinary and integral partnership between physicians, engineers, and computer scientists [26,30]. Therefore, it would be beneficial for future physicians to learn the fundamentals of AI in medical applications to comfortably work with AI technologies and meaningfully apply incoming technological innovations in medicine. It should be noted that certain residency programs, most prominently in radiology, have emphasized the requirement that their trainees have a knowledge of AI fundamentals [14,31-36]. This could be due to the higher prevalence of the use of imaging in AI relative to, for example, genomic data, which is only beginning to be studied with AI in medicine.

Our survey also showed that 91.5% (355/388) of surveyed US medical students agreed that training on AI concepts in medical school was important for their future careers (Figure 2) and 79.3% (308/388) were excited to use AI technologies (Figure 1). These opinions were not significantly affected by whether the respondents had prior formal AI training and indicate that current US medical students not only realize the need for incorporating AI topics into medical education as a “checkbox” to better prepare for future technological revolutions in medicine but are also enthusiastic about embracing such changes. This point is further reflected by our finding that 18.1% (70/386) of US medical students expressed hesitance in pursuing 1 of their top 3 desired specialties due to the incorporation of AI in that specialty (Multimedia Appendix 2). In contrast, other survey studies have found that 54% and 49% of medical students in Germany and Canada, respectively, were less likely to choose certain specialties due to the future incorporation of AI [19,22]. Most surveyed US medical students did not consider the inclusion of AI in medical education as a distraction but were instead excited to learn about AI in medicine, which was further demonstrated by their strong eagerness to explore AI in medical topics (Figure 1).

Furthermore, there is a lack of a structured approach to teaching AI systems in medical education. One of the questions our survey aimed to answer was what methodologies medical students prefer for learning about AI in health care (Figure 3). Our data showed that students preferred more medical student–directed and flexible opportunities to learn about AI in medicine, such as short lectures, formal preclinical electives, Q and A panels, and programming workshops. Currently, there is a lack of such opportunities; we found that students had obtained information on AI from other sources, including the media (263/386, 68.1%), family and friends (134/386, 34.7%), and online forums (98/386, 25.4%) (Multimedia Appendix 2). This finding is consistent with previous reports on where German medical students obtained their exposure to AI in medicine [21]. One example of curricular integration of AI is the University of Toronto Faculty of Medicine’s 14-month Computing for Medicine course, which began in 2019 [37]. However, the pace of change in medical education in adding AI-related topics is relatively slow compared to the pace at which the application of AI in medicine is currently progressing [38]. Thus, some leading experts have pushed for more radical changes in medical education or more extracurricular opportunities for students [17,26,29]. With the current shift of US medical education from strictly in-class learning to increased reliance on external resources (such as popular online learning platforms from Pathoma, Boards & Beyond, and Osmosis), as well as the advent of massive open online courses as a primary source of self-directed AI education, current medical students may be more receptive to self-directed learning based on extracurricular resources [39,40]. Thus, although most surveyed medical students preferred learning about AI through formal media, either formal curricular changes to incorporate AI should ramp up in pace or, as a potential alternative, online, freely accessible resources should be created for medical students to learn about AI [29].

Finally, it is important to distinguish between 2 sets of AI competencies. The first includes “core” competencies that most future physicians should know for their day-to-day practice, and the second includes “advanced” competencies for future physicians who intend to drive research and innovation in the field of AI in medicine [29]. While integration of AI topics into formal curricula may be sufficient for most medical students, research opportunities and mentorships should be provided specifically for future physician–scientists and innovators. Figures 3 and 4 show potential areas of AI topic concentration that differ between the 2 groups. Topics such as “fundamental concepts of AI,” “strengths and weaknesses of AI,” and “ethics of AI” were deemed interesting by both groups of respondents (ie, ≤2 hours vs ≥3 hours per month preferred for studying AI). Topics such as “translational science,” “global health and AI,” and “AI in medical research” can be directed specifically toward medical students who wish to go beyond the minimum required knowledge for future physicians regarding AI topics. To deliver these 2 sets of AI competencies, different resources should be employed for each group of learners. For example, our study showed that programming workshops, research teams, and conferences should be created that are tailored to the “advanced” learners, while short lectures would be highly valuable for both sets of learners.

### Limitations

Our study is admittedly not without limitations. First, although it agrees with results from other nations, our results do not fully represent the entire US medical student body due to a small sample size. Moreover, there might have been selection bias, because the respondents might have been students who were particularly interested in AI, especially considering that there was no financial incentive for survey completion. Finally, we did not analyze the importance of adding AI topics to the medical school curriculum in the context of other, already existing, medical school curricula, and we thus did not gauge the relative importance of AI topics.

### Conclusion

A large majority of current surveyed US medical students recognized the important role of AI in medicine and expressed excitement to learn more about AI fundamentals and applications in medicine. Nonetheless, only a minority of the students had knowledge of AI and medicine. The surveyed students were excited to learn about this topic and preferred formal, yet flexible, ways to approach AI in medical schools. However, currently available resources to learn about AI-related topics are limited in most US medical schools. Based on our work and prior surveys in other nations, we highlight the acute need to incorporate AI-related topics in the medical school curriculum.

## Appendix

Multimedia Appendix 1 Qualtrics survey assessing perceptions of US medical students on AI education.

Multimedia Appendix 2 Detailed answers to survey questions.

## Abbreviations

AI: artificial intelligence
