# Supplementary material for: Perceptions of US Medical Students on Artificial Intelligence in Medicine: Mixed Methods Survey Study
Source: JMIR Med Educ. 2022 Oct 21;8(4):e38325. doi: 10.2196/38325 (PMC9636531; doi:10.2196/38325)
Supplement: Multimedia Appendix 1 [file mededu_v8i4e38325_app1.pdf]

CONSENT WAIVER PAGE

Thank you for being willing to participate in this survey! The data you provide in this survey will be very valuable, and will be used to establish the necessity and guide the future of medical undergraduate education on the topic of AI in medicine. Below is the consent form. After reading it, if you decide to participate in this survey, click the "Yes, I consent" button at the end of the survey, then click the next button.

Department of Physiology and Pharmacology

3000 Arlington Avenue,

Toledo OH 43614

1-800-586-5336

ADULT RESEARCH SUBJECT - INFORMED CONSENT FORM

The Perceptions of US Medical Students on Artificial Intelligence (AI) In Medicine: a Nationwide Survey

Principal Investigator: Xi Cheng PhD, 419-383-4076

Other Investigators: David Liu, 401-662-8518

Purpose: You are invited to participate in the research project entitled ”The Perceptions of US Medical Students on Artificial Intelligence (AI) In Medicine: a nationwide survey” which is being conducted at the University of Toledo College of Medicine and Life Sciences under the direction of Dr. Xi Cheng and David Liu. The purpose of this study is to assess the attitudes US medical students have towards AI’s role in medicine, as well as their current familiarity and knowledge with the subject. In addition, this study aims to assess the interest US medical students have in gaining more exposure to information regarding AI, and through what means they prefer this information to be delivered.

Description of Procedures: This research survey will take place digitally via an online survey that will be sent, nationwide, to US schools of allopathic and osteopathic medicine for their respective student bodies to complete. The anonymous survey is

estimated to take approximately 5 minutes. The platform used to conduct the survey is called qualtricsXM. You are asked to complete four parts of the survey. The data collected will be evaluated, analyzed, and included in a research paper assessing the perceptions of US medical students on the topic of AI in medicine. In addition, after the completion of the survey, we will provide an email address you can use to contact us for more opportunities specific for medical students to explore the topic of AI in medicine. If you do decide to contact us and provide your information, it will remain separated from your survey responses. Dr. Xi Cheng PhD and Dr. Safwan Halabi MD of Stanford University helped in guiding the survey creation process, specifically advising the research team on what questions should we include or discard in the survey.

Potential Risks: The survey is completely anonymous with no protected health information (PHIs) nor personal identifiers which reduces the risks of losing confidentiality or anonymity. The survey includes sections that have minimal harmful or negative impact on your physical, psychological, emotional, or social/economic well-being. There are no legal risks involved as well. In order to minimize any discomfort or other hidden risks for you, the survey has been reviewed and edited by multiple medical students, the principal investigator and a board-licensed radiologist.

Potential Benefits: The only direct benefit to you if you participate in this research may be that you will learn about how surveys are run and you may learn more about the role of AI in the field of medicine. The field of medicine may benefit from this research by using these findings as a basis for medical schools to provide meaningful avenues to expose students to the topic of AI. The end goal of this research is to guide the future of undergraduate medical education in response to the incorporation of AI into medical practice

Confidentiality: This survey will be administered via the reputable third party service, qualtricsXM. No identifying information will be collected through this survey. There are no PHIs nor identifiers of any kind. The data will be researched and analyzed by only the principal investigator and the members of the research team.

Below is a statement from the Qualtrics' website commenting on the security of their program:
"Qualtrics' most important concern is the protection and reliability of customer data. Our servers are protected by high-end firewall systems and scans are performed regularly to ensure that any vulnerabilities are quickly found and patched."

Voluntary Participation: The information collected from you may be de-identified and used for future research purposes. As a reminder, your participation in this research is voluntary. Your refusal to participate in this study will involve no penalty or loss of

benefits to which you are otherwise entitled and will not affect your relationship with The University of Toledo or any of your classes or credits. You may skip any questions that you may be uncomfortable answering. In addition, you may discontinue participation at any time without any penalty or loss of benefits.

Contact Information: If you have any questions at any time before, during or after your participation or experience any distress you should contact a member of the research team below:
Principal Investigator: Dr. Xi Cheng PhD, 419-383-4076
Co-investigator: David Liu, 401-662-8518

If you have questions beyond those answered by the research team or your rights as a research subject or research-related injuries, the Chairperson of the SBE Institutional Review Board may be contacted through the Human Research Protection Program on the main campus at (419) 530-6167.

CONSENT SECTION – Please read carefully

You are making a decision whether or not to participate in this research study. Clicking "Yes, I consent" and completing the survey will act as your consent since no name or any other identifiable data are collected. You indicate that you have read the information provided above, you have had all your questions answered, and you have decided to take part in this research.
By participating in this research, you confirm that you are at least 18 years old.

- ○ Yes, I consent
- ○ No, I do not consent (will end survey)

DEMOGRAPHICS

DEMOGRAPHICS
(Page 1/4)

Are you an MD or a DO student?

- MD
- DO

Select your medical school (full school name listed):

Select your medical school:

Please select your current year in medical school.

What is your age?

What is your first choice specialty?

What is your second choice specialty?

What is your third choice specialty?

[dropdown]

Did you receive formal education (college or university courses) on AI/ML topics?

- Yes
- No

Block 2

SURVEYING CURRENT PERCEPTIONS OF ARTIFICIAL INTELLIGENCE (AI) IN MEDICINE (Page 2/4)

These next 13 short questions will assess what you think about AI in medicine. We believe this section to be the most important part of the survey.
Please skip the answer only if you truly feel unsure about any other answer options.

Please rate how strongly you agree or disagree with the statements below:

	Strongly disagree	Somewhat disagree	Neither agree nor disagree	Somewhat agree	Strongly agree
AI will take on a significant role in medicine during my lifetime	O	O	O	O	O
I am excited about using AI tech as a future physician	O	O	O	O	O
I understand AI concepts like: CNN, cross validation, ROC AUC, hyperparameters, deep learning, hidden layers, etc.	O	O	O	O	O
I can list some examples of recent clinically-relevant AI research	O	O	O	O	O

	Strongly disagree	Somewhat disagree	Neither agree nor disagree	Somewhat agree	Strongly agree
I can list the strengths/benefits of using AI in medicine	○	○	○	○	○
I can list the weaknesses/pitfalls of using AI in medicine	○	○	○	○	○
It's hard to understand and approach AI because of media sensationalism.	○	○	○	○	○
I can separate "hype" AI articles vs. clinically-relevant AI articles	○	○	○	○	○
I am worried about the ethics of using AI in medicine	○	○	○	○	○
Some training on AI concepts and related topics during medical school can be useful for my future career	○	○	○	○	○
My school offers resources if I want to explore the topic of AI in medicine	○	○	○	○	○
Learning the relevant topics of AI in medicine will significantly detract me from my medical school curriculum. (Topics include: ethics, criticizing AI articles, pros/cons of AI, job security, etc.)	○	○	○	○	○
I want to learn what medical students should know about AI in medicine (scroll down and click the next button to continue after answering)	○	○	○	○	○

SURVEYING CURRENT PERCEPTIONS OF AI IN MEDICINE

(Page 3/4)

Which specialties do you think will be most affected by AI? (Please select the top three):

- ☐ Anesthesiology
- ☐ Child Neurology
- ☐ Dermatology

- ☐ Emergency Medicine
- ☐ Family Medicine
- ☐ General Surgery
- ☐ Internal Medicine
- ☐ Interventional Radiology
- ☐ Neurosurgery
- ☐ Neurology
- ☐ Obstetrics and Gynecology
- ☐ Ophthalmology
- ☐ Orthopedic Surgery
- ☐ Otolaryngology
- ☐ Pathology
- ☐ Pediatrics
- ☐ Physical Medicine and Rehabilitation
- ☐ Plastic Surgery
- ☐ Psychiatry
- ☐ Radiology (Diagnostic)
- ☐ Radiation Oncology
- ☐ Urology
- ☐ Vascular Surgery

I am less likely to choose these selected specialties because of the anticipated integration of AI.

- ○ Strongly disagree
- ○ Disagree
- ○ Somewhat disagree
- ○ Neither agree nor disagree
- ○ Somewhat agree

- Agree
- Strongly agree

Where did you gain your exposure to AI? (Please select all that apply):

- Media (television, YouTube, Twitter)
- Online forums
- Peer-reviewed articles
- Formal lectures
- Professors/doctors
- Research projects
- Books
- Conferences
- Family and Friends
- ______ Other:

What do students want to know?

SURVEYING INTEREST
(Page 4/4)

What are some ways that you think would be MOST USEFUL for your medical school to offer to help students like you to explore the topic of AI in medicine? (Please select all that apply):

- Q&A panels with leaders in the field
- Symposiums where experts present and discuss their AI-related research
- Short lectures (3-5 total hours) on the fundamentals of AI in medicine
- Student-led journal club on AI articles
- Interdisciplinary research teams with the goal of publication

- ☐ Incentivization to go to AI conferences
- ☐ Formal preclinical elective
- ☐ Collation of various good online resources into a single location
- ☐ Workshops on programming AI models
- ☐ [ ] Other: (list your idea here)

What are some specific topics within AI in medicine you would MOST be interested in? (Please select all that apply):

- ☐ Strengths and weaknesses of using AI in medicine
- ☐ When to use AI in medicine?
- ☐ Fundamental concepts of AI
- ☐ Types of models in AI (NLP, CNNs, etc.)
- ☐ How to create AI models using Python
- ☐ Most recent and significant AI health innovations/research in my top 3 listed specialties
- ☐ Ethics of AI
- ☐ What aspects of a physician's job can be replaced with AI and which can't?
- ☐ How to critique AI articles
- ☐ How to bring AI models to clinical practice (translational science)
- ☐ AI in medical research
- ☐ Roles of individuals in multidisciplinary teams doing AI research
- ☐ Global health implications of AI
- ☐ Effects of AI on health inequalities
- ☐ [ ] Other: (list your idea here)

The maximum amount of time I would like to spend exploring the topic of AI in medicine <u>per month</u> is...

- ○ None

- 30 minutes
- 1 hour
- 2 hours
- 3 hours
- 4 hours
- 5 hours or more

Please use this space to share any other thoughts on the topic that you feel are significant but weren’t completely captured in the survey.

If you are interested in the topic of AI in medicine, please don't click away yet at the next page! There are exciting opportunities in the near future specifically for you!

This is the end of the survey. Clicking the next button will submit the survey.

Powered by Qualtrics
