# Supplementary material for: Perceptions of US Medical Students on Artificial Intelligence in Medicine: Mixed Methods Survey Study
Source: JMIR Med Educ. 2022 Oct 21;8(4):e38325. doi: 10.2196/38325 (PMC9636531; doi:10.2196/38325)
Supplement: Multimedia Appendix 2 [file mededu_v8i4e38325_app2.docx]

## **Supplementary Data**

**Supplementary Figure 1: Qualtrics Survey PDF**

**Supplementary Table 1: Answers to Qualtrics Survey**

| **“Which specialties do you think will be most affected by AI? (Select the top three)” n = 386** | | | |  |
| --- | --- | --- | --- | --- |
|  | **Answer** | **%*** | **Count** |  |
|  | Anesthesiology | 18.4% | 71 |  |
|  | Child Neurology | 2.1% | 8 |  |
|  | Dermatology | 10.1% | 39 |  |
|  | Radiology (Diagnostic) | 72.0% | 278 |  |
|  | Emergency Medicine | 15.3% | 59 |  |
|  | Family Medicine | 10.6% | 41 |  |
|  | General Surgery | 17.1% | 66 |  |
|  | Internal Medicine | 16.3% | 63 |  |
|  | Interventional Radiology | 24.6% | 95 |  |
|  | Neurosurgery | 15.0% | 58 |  |
|  | Neurology | 8.5% | 33 |  |
|  | Obstetrics and Gynecology | 1.6% | 6 |  |
|  | Ophthalmology | 5.7% | 22 |  |
|  | Orthopedic Surgery | 5.7% | 22 |  |
|  | Otolaryngology | 0.5% | 2 |  |
|  | Pathology | 43.3% | 167 |  |
|  | Pediatrics | 1.0% | 4 |  |
|  | Physical Medicine and Rehabilitation | 3.4% | 13 |  |
|  | Plastic Surgery | 2.6% | 10 |  |
|  | Psychiatry | 1.6% | 6 |  |
|  | Radiation Oncology | 19.4% | 75 |  |
|  | Urology | 1.0% | 4 |  |
|  | Vascular Surgery | 4.1% | 16 |  |
| **“I am less likely to choose these specialties because of the anticipated integration of AI” n = 386** | | | |  |
|  | **Answer** | **%** | **Count** |  |
|  | Strongly disagree | 26.68% | 103 |  |
|  | Disagree | 28.50% | 110 |  |
|  | Somewhat disagree | 8.55% | 33 |  |
|  | Neither agree nor disagree | 18.13% | 70 |  |
|  | Somewhat agree | 12.18% | 47 |  |
|  | Agree | 3.63% | 14 |  |
|  | Strongly agree | 2.33% | 9 |  |
| **“Where did you gain your exposure to AI? (Please select all that apply)” n = 386** | | | |  |
|  | **Answer** | **%*** | **Count** |  |
|  | Media (television, YouTube, Twitter) | 68.1% | 263 |  |
|  | Family and Friends | 34.7% | 134 |  |
|  | Online forums | 25.4% | 98 |  |
|  | Professors/doctors | 23.1% | 89 |  |
|  | Research projects | 20.2% | 78 |  |
|  | Peer-reviewed articles | 18.1% | 70 |  |
|  | Books | 15.0% | 58 |  |
|  | Formal lectures | 14.2% | 55 |  |
|  | Conferences | 8.3% | 32 |  |
|  | Other: | 6.5% | 25 |  |
|  | Total |  | 386 |  |
| **“What are some ways that you think would be MOST USEFUL for your medical school to offer to help students like you to explore the topic of AI in medicine? (Please select all that apply)” n = 378** | | | | |
|  | **Answer** | **%*** | **Count** | ***P*-value (≤2 hours vs ≥3 hours per month to learn AI)** |
|  | Short lectures (3-5 total hours) on the fundamentals of AI in medicine | 69.8% | 264 | .06 |
|  | Formal preclinical elective | 47.6% | 180 | .06 |
|  | Q&A panels with leaders in the field | 44.2% | 167 | .24 |
|  | Workshops on programming AI models | 43.4% | 164 | <.001 |
|  | Collation of various good online resources into a single location | 38.4% | 145 | .87 |
|  | Symposiums where experts present and discuss their AI-related research | 36.0% | 136 | .01 |
|  | Interdisciplinary research teams with the goal of publication | 23.8% | 90 | .01 |
|  | Incentivization to go to AI conferences | 22.8% | 86 | <.001 |
|  | Student-led journal club on AI articles | 20.1% | 76 | .13 |
|  | Other: (list your idea here) | 1.3% | 5 | .66 |
| **“What are some specific topics within AI in medicine you would MOST be interested in? (Please select all that apply)” n = 379** | | | | |
|  | **Answer** | **%*** | **Count** | ***P*-value (≤2 hours vs ≥3 hours per month to learn AI)** |
|  | Fundamental concepts of AI | 65.2% | 247 | .99 |
|  | When to use AI in medicine? | 59.9% | 227 | .96 |
|  | Strengths and weaknesses of using AI in medicine | 59.1% | 224 | .71 |
|  | Ethics of AI | 55.7% | 211 | .29 |
|  | What aspects of a physician’s job can be replaced with AI and which can’t? | 53.6% | 203 | .15 |
|  | Effects of AI on health inequalities | 47.2% | 179 | .08 |
|  | How to bring AI models to clinical practice (translational science) | 45.6% | 173 | .01 |
|  | AI in medical research | 42.0% | 159 | .04 |
|  | Types of models in AI (NLP, CNNs, etc.) | 41.2% | 156 | <.001 |
|  | Global health implications of AI | 39.3% | 149 | .01 |
|  | Most recent and significant AI health innovations/research in my top 3 listed specialties | 39.1% | 148 | .03 |
|  | How to create AI models using Python | 30.6% | 116 | <.001 |
|  | How to critique AI articles | 23.7% | 90 | .09 |
|  | Roles of individuals in multidisciplinary teams doing AI research | 11.9% | 45 | .03 |
|  | Other: (list your idea here) | 0.3% | 1 | .76 |
| **“The maximum amount of time I would like to spend exploring the topic of AI in medicine per month is...” n = 379** | | | |  |
|  | **Answer** | **%** | **Count** |  |
|  | None | 2.90% | 11 |  |
|  | 30 minutes | 12.66% | 48 |  |
|  | 1 hour | 26.39% | 100 |  |
|  | 2 hours | 25.59% | 97 |  |
|  | 3 hours | 11.35% | 43 |  |
|  | 4 hours | 10.82% | 41 |  |
|  | 5 hours or more | 10.29% | 39 |  |

*Percentages do not add up to 100% because this calculates the percentage of students that picked this answer and students can pick more than one answer

**Supplementary Table 2: Choice of specialties (n = 390)**

|  | **First choice** | | **Second choice** | | **Third choice** | |
| --- | --- | --- | --- | --- | --- | --- |
| **Specialty** | % | Count | % | Count | % | Count |
| Anesthesiology | 3.33% | 13 | 4.87% | 19 | 3.85% | 15 |
| Child Neurology | 1.54% | 6 | 1.54% | 6 | 0.00% | 0 |
| Dermatology | 1.79% | 7 | 1.28% | 5 | 2.31% | 9 |
| Radiology (Diagnostic) | 4.87% | 19 | 3.08% | 12 | 2.31% | 9 |
| Emergency Medicine | 9.74% | 38 | 6.15% | 24 | 5.13% | 20 |
| Family Medicine | 6.67% | 26 | 10.00% | 39 | 11.28% | 44 |
| General Surgery | 4.62% | 18 | 8.72% | 34 | 3.85% | 15 |
| Internal Medicine | 15.13% | 59 | 14.10% | 55 | 11.03% | 43 |
| Interventional Radiology | 0.51% | 2 | 2.31% | 9 | 2.05% | 8 |
| Neurosurgery | 2.05% | 8 | 1.54% | 6 | 0.77% | 3 |
| Neurology | 3.59% | 14 | 2.31% | 9 | 3.59% | 14 |
| Obstetrics and Gynecology | 5.90% | 23 | 3.33% | 13 | 4.62% | 18 |
| Ophthalmology | 2.56% | 10 | 2.05% | 8 | 1.28% | 5 |
| Orthopedic Surgery | 6.92% | 27 | 3.08% | 12 | 3.59% | 14 |
| Otolaryngology | 1.79% | 7 | 0.51% | 2 | 1.54% | 6 |
| Pathology | 1.03% | 4 | 0.51% | 2 | 0.77% | 3 |
| Pediatrics | 6.41% | 25 | 7.44% | 29 | 4.87% | 19 |
| Physical Medicine and Rehabilitation | 2.31% | 9 | 2.82% | 11 | 2.56% | 10 |
| Plastic Surgery | 1.28% | 5 | 2.05% | 8 | 1.28% | 5 |
| Psychiatry | 4.10% | 16 | 4.62% | 18 | 4.10% | 16 |
| Radiation Oncology | 1.79% | 7 | 1.54% | 6 | 1.28% | 5 |
| Urology | 1.03% | 4 | 0.77% | 3 | 0.51% | 2 |
| Vascular Surgery | 0.77% | 3 | 1.03% | 4 | 0.77% | 3 |
| Other (unlisted) | 3.08% | 12 | 3.08% | 12 | 4.36% | 17 |

**Supplementary Table 3: Statements about AI in medicine (Likert scale).**

| **Statement** | **1** | **N** | **2** | **N** | **3** | **N** | **4** | **N** | **5** | **N** | **Total** | ***P*-values (formal education in AI vs no formal education)** | **Cramer’s V (if *P* <.05)** |
| --- | --- | --- | --- | --- | --- | --- | --- | --- | --- | --- | --- | --- | --- |
| AI will take on a significant role in medicine during my lifetime | 1.28% | 5 | 3.59% | 14 | 5.13% | 20 | 42.82% | 167 | 47.18% | 184 | 390 | .15 |  |
| I am excited about using AI tech as a future physician | 1.55% | 6 | 6.70% | 26 | 12.37% | 48 | 39.43% | 153 | 39.95% | 155 | 388 | .08 |  |
| I am worried about the ethics of using AI in medicine | 3.87% | 15 | 15.46% | 60 | 19.33% | 75 | 45.88% | 178 | 15.46% | 60 | 388 | .96 |  |
| I can list some examples of recent clinically-relevant AI research | 45.62% | 177 | 23.20% | 90 | 8.25% | 32 | 17.27% | 67 | 5.67% | 22 | 388 | <.001 | .29 |
| I can separate “hype” AI articles vs. clinically-relevant AI articles | 14.43% | 56 | 27.32% | 106 | 19.07% | 74 | 32.99% | 128 | 6.19% | 24 | 388 | <.001 | .26 |
| I understand AI concepts like: CNN, cross validation, ROC AUC, etc. | 59.02% | 229 | 22.16% | 86 | 4.90% | 19 | 10.05% | 39 | 3.87% | 15 | 388 | <.001 | .35 |
| It's hard to understand and approach AI because of media sensationalism. | 5.43% | 21 | 19.38% | 75 | 32.04% | 124 | 35.66% | 138 | 7.49% | 29 | 387 | .92 |  |
| I want to learn what medical students should know about AI in medicine | 0.77% | 3 | 3.35% | 13 | 6.44% | 25 | 43.30% | 168 | 46.13% | 179 | 388 | .73 |  |
| Learning the relevant topics of AI in medicine will significantly detract me from my medical school curriculum. | 22.94% | 89 | 43.56% | 169 | 18.04% | 70 | 12.89% | 50 | 2.58% | 10 | 388 | .02 | .12 |
| My school offers resources if I want to explore the topic of AI in medicine | 32.04% | 124 | 33.33% | 129 | 25.84% | 100 | 7.75% | 30 | 1.03% | 4 | 387 | .06 |  |
| Some training on AI concepts and related topics during medical school can be useful for my future career | 0.77% | 3 | 1.55% | 6 | 6.19% | 24 | 43.30% | 168 | 48.20% | 187 | 388 | .57 |  |
| I can list the strengths/benefits of using AI in medicine | 8.76% | 34 | 18.30% | 71 | 16.24% | 63 | 44.33% | 172 | 12.37% | 48 | 388 | <.001 | .22 |
| I can list the weaknesses/pitfalls of using AI in medicine | 8.25% | 32 | 19.85% | 77 | 14.69% | 57 | 44.85% | 174 | 12.37% | 48 | 388 | <.001 | .23 |

Note. 1 = Strongly disagree, 2 = somewhat disagree, 3 = neither agree nor disagree, 4 = somewhat agree, 5 = strongly agree

**Supplementary Table 4: Free response section**

| \| 1. The ethics of AI are of most interest to me. \| \| --- \| \| 1. I feel like I really didn't know at all about AI in medicine and hope there will be educational opportunities in the future (residency programs in addition to medical school) for those of us who will be graduating before changes are made to medical education curriculum. \| \| 1. I honestly have heard very little about the subject. \| \| 1. AI is interesting and will be Incorporated into medical practices more and more through our lifetimes. But I don’t think it’s necessary for pre-clinical medical students to understand how to utilize AI. I feel that it’s more important to learn about AI in residency versus medical school \| \| 1. I think AI is groundbreaking and has already had a significant impact on my research in the first year of my Ph.D. I've reviewed and been featured as a co-author on AI-driven peer-reviewed papers and had to present them in journal clubs. While I understand the surface concepts of AI and how to rank the impact of different AI-based publications, I have never directly created an AI model myself which I believe is a valuable skill to have. I feel like I'm behind in my understanding of basic computer science and wish I took more courses about it in college. I'm also a little intimidated by learning a coding langue like python. That being said, I'm motivated to acquire these skills as I complete my thesis work. \| \| 1. I am against adding more components to preclinical medical education. It is a low-effort suggestion that is pitched for everything. Preclinical education is zero-sum. \| \| 1. One Book recommendation for this exact topic is “Deep Medicine” by Eric Topol which influenced a lot of my survey results and perspectives on AI in healthcare \| \| 1. pros and cons learning about for sure. How and when to use it. Learning new technologies being used. Ethics and health equity applications. \| \| 1. Interesting topic, but at this point in my career I don't care to spend any time on it, I have more important things to spend my time on. \| \| 1. I think that AI has great promise for improving healthcare, but I am worried about the negative effects it might have (job loss, doctors becoming over-reliant on it, etc.) \| \| 1. The disruption that AI may have on physician careers is very concerning to me. On the one hand, I am excited about the prospect of AI being able to reduce medical errors, increase diagnostic accuracy, and better patient health. On the other hand, I have read many articles about the potential for AI to replace countless jobs across numerous sectors, including doctors. After spending many years training and taking on significant debt, I am very concerned about this potential job replacement. I don't think that physicians should try to impede technological progress to save their own career but I do think it's imperative that physicians play a central role in the public and institutional discourse about the proper role of AI versus human roles. If we don't take the lead, someone else will, and they may not have our or our patients best interests at heart. While the future is hazy as always, I would love for my school would provide us with foundational knowledge that will help us navigate these important issues throughout our careers. \| \| 1. I don't think medical students have enough computer science and engineering background to learn much about AI \| \| 1. This an extremely important topic that needs more focus. \| \| 1. I am getting a PhD in computational biology, and I will be spending a serious amount of time on machine learning and AI during that PhD. \| \| 1. I know very little about computer science, and indeed many of my medical school peers know just as little as I. However, we generally recognize the potential benefits of this field of research and are thus more inclined to wonder about its utility rather than worry about it "stealing our jobs." If it can help the patient (even at the cost of some of the traditional roles of a physician), it is worth pursuing in my opinion. \| \| 1. Especially interested in incorporating AI into and pulling requisite data from EMR, both for research and practical clinical applications. \| \| 1. No more than 15 minutes per month of time should be spent on teaching about this topic to medical students. It should not be a significant part of our education at this stage, but certainly should be present. \| \| 1. I would like to be involved \| \| 1. I think you had plenty of answer options to capture my thoughts! \| \| 1. I would like to emphasize my intrigue in learning more about the ethics behind utilizing AI in medicine. I feel that this is one of the main barriers, if not the largest barrier to increasing AI's utilization in medicine. \| \| 1. One unresolved problem is medicine how to safely integrate AI products into clinical practice. There are currently no standards that ensure AI products are robust when generalizing to new patient populations. \| \| 1. As a person who is not inherently involved in Machine Learning but is surrounded by people in various CS fields (not related to medical applications), I feel as though it isn't terribly necessary for medical students to fully grasp all the fundamentals of AI nor for them to have programming workshops (I would speak differently for doctors who can immediately and readily apply what they learn into practice in an efficacious way). That said, I do believe that the incorporation of AI into how we interface with medical technology is inevitable, and it would be a great disservice for people walking into the field to be unfamiliar with the implications and applications of AI. To that end, I feel as though understanding the potential of AI within medicine, as well as the pro/cons and relevant ethics should be the first thing we consider incorporating into the medical education, with all else being an elective that those who are interested can further explore. \| \| 1. Being involved in AI research is a must for me. \| \| 1. Ethics of AI was mentioned several times in this survey; however, I feel that there wasn't a clear distinction whether this was referring to ethics of treating patients with AI vs ethical treatment of AI itself. \| |
| --- | --- | --- | --- | --- | --- | --- | --- | --- | --- | --- | --- | --- | --- | --- | --- | --- | --- | --- | --- | --- | --- | --- | --- | --- |
